# Cyclin-dependent kinase inhibitor dinaciclib potently synergizes with cisplatin in preclinical models of ovarian cancer

**DOI:** 10.18632/oncotarget.3717

**Published:** 2015-03-30

**Authors:** Xiu-Xiu Chen, Feng-Feng Xie, Xiu-Jie Zhu, Feng Lin, Shi-Shi Pan, Li-Hua Gong, Jian-Ge Qiu, Wen-Ji Zhang, Qi-Wei Jiang, Xiao-Long Mei, You-Qiu Xue, Wu-Ming Qin, Zhi Shi, Xiao-Jian Yan

**Affiliations:** ^1^ Department of Gynecology, The First Affiliated Hospital of Wenzhou Medical University, Wenzhou, Zhejiang, China; ^2^ Department of Cell Biology and Institute of Biomedicine, National Engineering Research Center of Genetic Medicine, Guangdong Provincial Key Laboratory of Bioengineering Medicine, College of Life Science and Technology, Jinan University, Guangzhou, Guangdong, China

**Keywords:** ovarian cancer, dinaciclib, cisplatin, combination therapy

## Abstract

Ovarian cancer is one of the most lethal of woman cancers, and its clinical therapeutic outcome currently is unsatisfied. Dinaciclib, a novel small molecule inhibitor of CDK1, CDK2, CDK5 and CDK9, is assessed in clinical trials for the treatment of several types of cancers. In this study, we investigated the anticancer effects and mechanisms of dinaciclib alone or combined with cisplatin in ovarian cancer. Dinaciclib alone actively induced cell growth inhibition, cell cycle arrest and apoptosis with the increased intracellular ROS levels, which were accompanied by obvious alterations of related proteins such as CDKs, Cyclins, Mcl-1, XIAP and survivin. Pretreatment with N-acety-L-cysteine significantly blocked ROS generation but only partially rescued apoptosis triggered by dinaciclib. Moreover, the combination of dinaciclib with cisplatin synergistically promoted cell cycle arrest and apoptosis, and inhibited the subcutaneous xenograft growth of ovarian cancer in nude mice. Altogether, dinaciclib potently synergizes with cisplatin in preclinical models of ovarian cancer, indicating this beneficial combinational therapy may be a promising strategy for treatment of ovarian cancer.

## INTRODUCTION

Ovarian cancer currently is the second most common gynecologic cancer in the worldwide with a low 5-years survival rate, approximately 44% [1]. Cytoreductive surgery with platinum-based chemotherapy was the preferred treatment for the patients with ovarian cancer. Despite of treatment with standard strategies, at least 60% of patients suffers the recurrent of ovarian cancer [2]. Therefore, development of novel therapeutic agents against ovarian cancer is an urgent necessity.

Dysregulation of cell cycle progression and aberrant activation of cyclin-dependent kinases (CDKs) are hallmarks of almost all human cancers [3, 4]. Amplification of CDKs, overexpression of cyclins, inactivation of (CDK inhibitors) CKIs, loss of retinoblastoma (Rb) expression all occur frequently in human malignancies [5, 6]. The CDKs are a well-characterized family of serine/threonine kinases, playing cardinal roles in regulating cell cycle progression by phosphorylation of Rb and other substrates [7]. Until now, eleven CDKs have been identified [8]. Cell cycle progression requires the activity of CDK1, CDK2, CDK4 and CDK6. CDK1-cyclin A/B, CDK2-cyclin A/E and CDK4/6-cyclin D complexes are responsible for driving cells through G2/M, G1/S transition and G1 phase, respectively. The irreversible arrest of cell cycle by stresses finally leads cells to apoptosis [9]. Except cell cycle regulation, the CDK family also has other functions. For instance, CDK5 is important for neuronal development [10], CDK7 is an integral component of the transcription factor TFIIH [11], and CDK9 is involved in the initiation and elongation steps of transcription [12-14]. Given the critical role of CDKs on regulating cell cycle and transcription processes which are commonly abnormal in cancer cells, targeting CDKs by small molecule inhibitors has been suggested as a potential therapy option for human cancers.

Dinaciclib (MK-7965, formerly SCH727965) is a potent and selective small molecule inhibitor of CDK2, CDK5, CDK1 and CDK9 with IC_50_ of 1 nM, 1 nM, 3 nM and 4 nM, respectively, and show better therapeutic index (TI: maximum tolerated dose/minimum effective dose; >10 *vs.* <1, respectively) than flavopiridol, the first CDKs inhibitor to enter the clinic trail [15]. Preclinical data have demonstrated that dinaciclib is active against a broad spectrum of human cancer cell lines with median IC_50_ of 11 nM by inducing cell cycle arrest and apoptosis [15]. The phase I clinical studies showed that dinaciclib administered at a dose of 0.33 mg/m^2^ as a 2-hour intravenous infusion on days 1, 8, 15 of a 28-day cycle was generally safe and well tolerated with the common adverse events including nausea, decreased appetite, anemia and fatigue [16]. The results of phase II study demonstrated that dinaciclib administered intravenous at the 50 mg/m^2^ dose was well tolerated, but without antitumor activity as monotherapy in patients with non-small cell lung cancer [17]. In addition, another phase II trial illustrated that dinaciclib at 50 mg/m^2^ administered as a 2-hour infusion every 21 days displayed some antitumor activity and was generally tolerated in patients with advanced breast cancer, but efficacy was not superior to capecitabine at 1250 mg/m^2^ administered orally twice daily in 21-day cycles [18]. Furthermore, dinaciclib administered at doses of 30-50 mg/m^2^ on day 1 of a 21-day cycle exhibited encouraging single-agent antitumor activity in patients with relapsed multiple myeloma [19]. Evaluation of dinaciclib in combination with other chemotherapeutical drugs for multiple types of cancers currently is in progress. In this study, we investigated that anticancer effects and mechanisms of dinaciclib alone or combined with cisplatin in preclinical models of ovarian cancer.

## RESULTS

### Dinaciclib inhibited the growth of ovarian cancer cells *in vitro*

**Figure 1:**
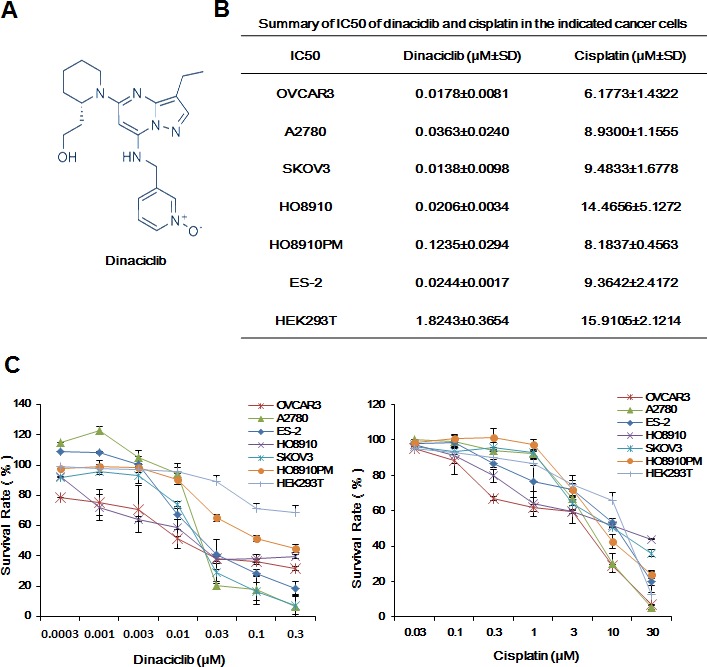
Dinaciclib inhibited the growth of ovarian cancer cells *in vitro* (**A**) Chemical structure of dinaciclib. (**B**) Summary of IC_50_ of dinaciclib and cisplatin in the indicated ovarian cancer cells was shown. Cells were grown in 96-well plates for 24 h and treated with the indicated concentrations of dinaciclib or cisplatin for 72 h. Cell survival was measured by MTT assay. The representative growth curves of cells treated with dinaciclib and cisplatin (**C**) are shown. Data were mean ±SD of three independent experiments.

To evaluate the effect of dinaciclib on ovarian cancer cells, cell survival was detected by MTT assay. Six human ovarian cancer cell lines were treated with increasing concentrations of dinaciclib for 72h. As shown in Figure 1, dinaciclib strongly inhibited ovarian cancer cells growth in a dose-dependent manner with the IC_50_ values range from 0.0138 to 0.1235μM. In addition, cisplatin also dose-dependently suppressed the growth of ovarian cancer cells with the IC_50_ of 6.1773 to 14.4656 μM. However, the IC_50_ of dinaciclib and cisplatin in human normal embryonic kidney HEK293T cells were 1.8243 μM and 15.9105 μM, respectively, suggesting dinaciclib is more cytotoxic to ovarian cancer cells than normal cells.

### Dinaciclib induced cell cycle arrest in ovarian cancer cells

**Figure 2:**
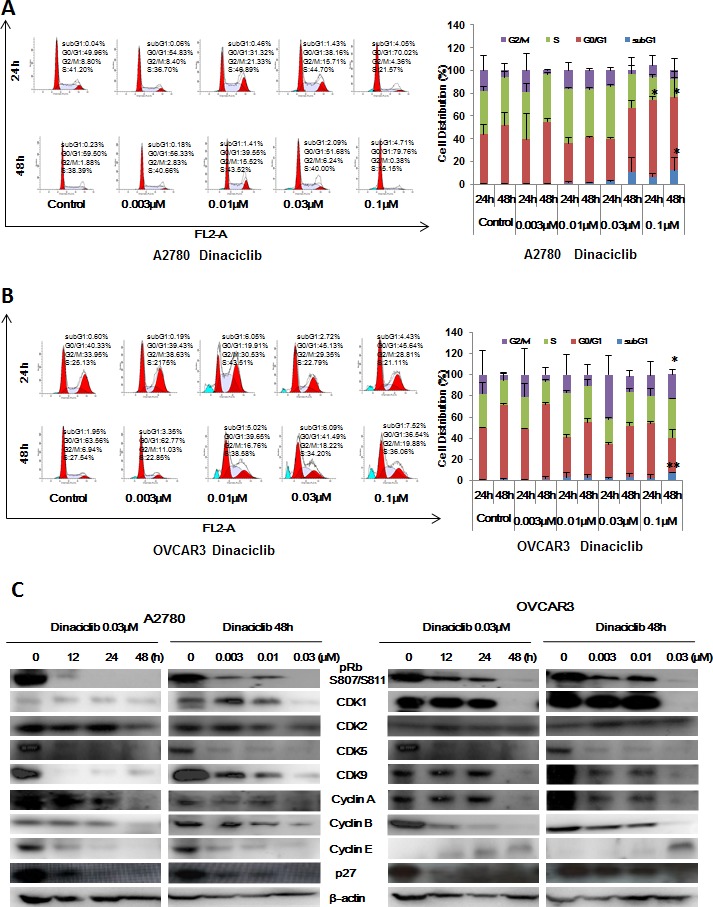
Dinaciclib induced cell cycle arrest in ovarian cancer cells A2780 (**A**) and OVCAR3 (**B**) cells were treated with dinaciclib at the indicated times and concentrations. The distribution of cell cycle was detected by FCM with PI staining. The percentages of subG1, G1/G0, S, G2/M phase were calculated using ModFit LT 3.0 software. The protein expression was examined by Western blot after lysing cells, and β-actin was used as loading control. The representative charts, quantified results and Western blot results (**C**) of three independent experiments were shown. *P < 0.05 and **P < 0.01 *vs.* corresponding control.

To explore whether the growth inhibition of ovarian cancer cells by dinaciclib is as a result of cell cycle arrest, cell cycle distribution was assessed after dinaciclib treatment. A2780 and OVCAR3 ovarian cancer cells were treated with dinaciclib (0.003, 0.01, 0.03 and 0.1 μM) for 24h and 48h, stained with PI and examined by FCM. The cell cycle distribution was analyzed with ModFit LT 3.0 software. Interestingly, dinaciclib had the different effects on the cell cycle distribution of A2780 and OVCAR3 cells, which enhanced the G2/M population at 0.01 μM and G0/G1 population at 0.1 μM in A2780 cells and the S population at 0.01 μM and G2/M population at 0.1 μM in OVCAR3 cells with the increase of subG1 population in both cells (Figure 2A and 2B). Next, the cell cycle related proteins were detected by Western blot to investigate the molecular mechanism of cell cycle arrest by dinaciclib. After treatment with dinaciclib, the protein levels of pRb S807/811, Cyclin A, Cyclin B, p27 and Cdk1, Cdk5, Cdk9, which are the targets of dinaciclib, were decreased in the time and dose dependent manners in both A2780 and OVCAR3 cells. However, the protein levels of another target of dinaciclib Cdk2 was decreased in A2780 cells but unchanged in OVCAR3 cells, and Cyclin E was decreased in A2780 cells but increased in OVCAR3 cells (Figure 2C).

### Dinaciclib induced apoptosis in ovarian cancer cells

**Figure 3:**
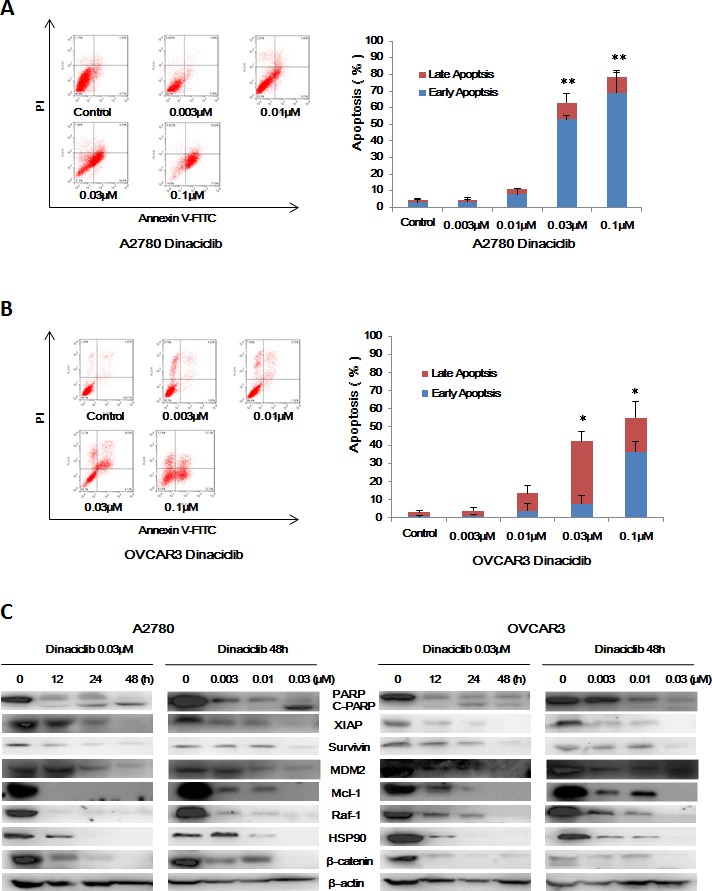
Dinaciclib induced apoptosis in ovarian cancer cells A2780 (**A**) and OVCAR3 (**B**) cells were treated with dinaciclib at the indicated time and concentrations. The apoptosis was detected by FCM Annexin V/PI staining. The proportions of Annexin V+/PI− and Annexin V+/PI+ cells indicated the early and late stage of apoptosis. The protein expression was examined by Western blot after lysing cells, and β-actin was used as loading control. The representative charts, quantified results and Western blot results (**C**) of three independent experiments were shown. *P < 0.05 and **P < 0.01 *vs.* corresponding control.

To determine whether the growth inhibition of ovarian cancer cells by dinaciclib is also due to apoptosis, cell apoptosis was assessed after dinaciclib treatment. A2780 and OVCAR3 cells were treated with dinaciclib (0.003, 0.01, 0.03 and 0.1 μM) for 48h, stained with Annexin V/PI and examined by FCM. As shown in Figure 3A and 3B, dinaciclib dose-dependently induced early apoptosis (Annexin V+/PI-) and late apoptosis (Annexin V+/PI+) in both cells. Futhermore, the apoptotic related proteins were detected by Western blot to investigate the molecular mechanism of cell apoptosis by dinaciclib. After treatment with dinaciclib, the cleaved PARP, which is the marker of apoptosis, was time- and dose-dependently generated in both cells. Furthermore, the protein levels of XIAP, survivin, MDM2, Mcl-1, Raf-1, HSP90 and β-catenin were significantly decreased in both cells (Figure 3C).

### ROS was critical for the anticancer effect of dinaciclib in ovarian cancer cells

**Figure 4:**
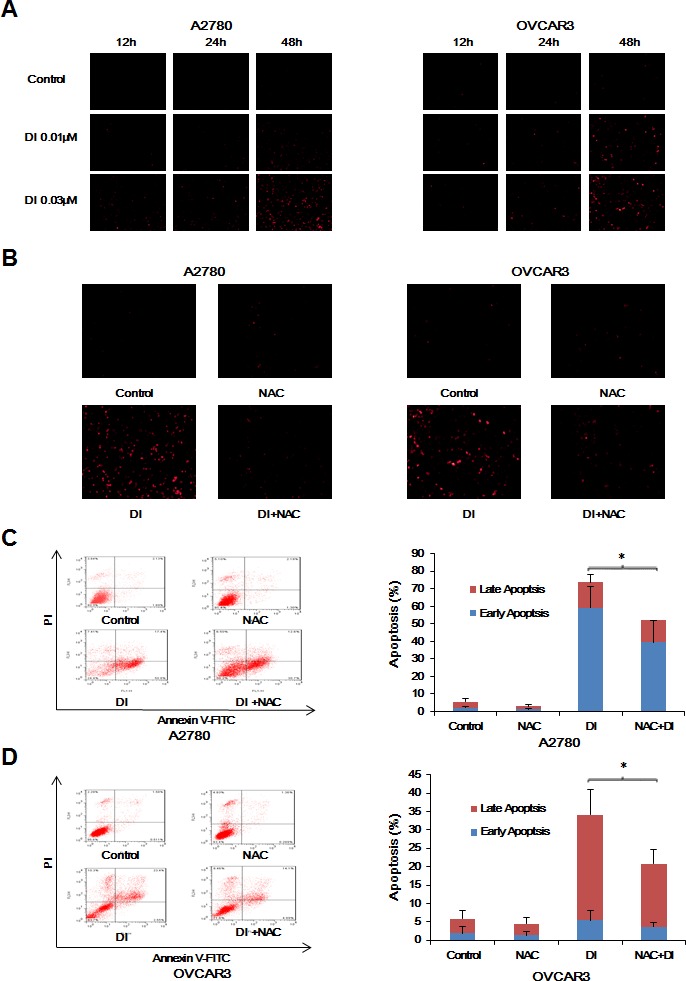
ROS was critical for the anticancer effect of dinaciclib in ovarian cancer cells A2780 and OVCAR3 cells were treated with dinaciclib at the indicated times and concentrations, stained with DHE and photographed under florescent microscope. The representative micrographs (**A**) of three independent experiments were shown. Cells were also treated with 0.03 μM dinaciclib for 48 h in the presence or absence of 5mM NAC pretreatment for 1h, stained with DHE and photographed under fluorescent microscope. The apoptosis was detected by FCM with Annexin V/PI staining. The proportions of Annexin V+/PI− and Annexin V+/PI+ cells indicated the early and late stage of apoptosis. The representative micrographs (**B**), charts and quantified results (**C**, **D**) of three independent experiments were shown. DI: Dinaciclib. *P < 0.05 and **P < 0.01 *vs.* corresponding control.

ROS plays an important role in tumorigenesis and chemotherapy of most anticancer drugs [20]. To assess the role of ROS in the anticancer effect of dinaciclib in ovarian cancer cells, we used dihydroethidium (DHE) as ROS fluorescent probe, which can be oxidized by ROS to oxide ethidium that binds with DNA to emit the detectable red fluorescence [21], to stain cells treated with dinaciclib. As shown in Figure 4A, dinaciclib enhanced the fluorescent signals of DHE in both A2780 and OVCAR3 cells in the concentration-and time-dependent manners, suggesting the intracellular ROS levels were enhanced after dinaciclib treatment. To further verify the relationship between dinaciclib induced apoptosis and ROS generation, both cells were treated with dinaciclib for 48h in the presence or absence of the antioxidative agent NAC pretreatment for 1h and stained with DHE. The dinaciclib-induced DHE fluorescent signals were totally reversed by NAC in both cells (Figure 4B). Moreover, cell apoptosis was detected by FCM with Annexin V/PI staining. The dinaciclib-induced apoptosis were partially blocked by NAC in both cells (Figure 4C and 4D), suggesting dinaciclib can induce both ROS dependent and independent apoptosis.

### Dinaciclib synergized with cisplatin to inhibit the growth of ovarian cancer cells *in vitro*

**Figure 5:**
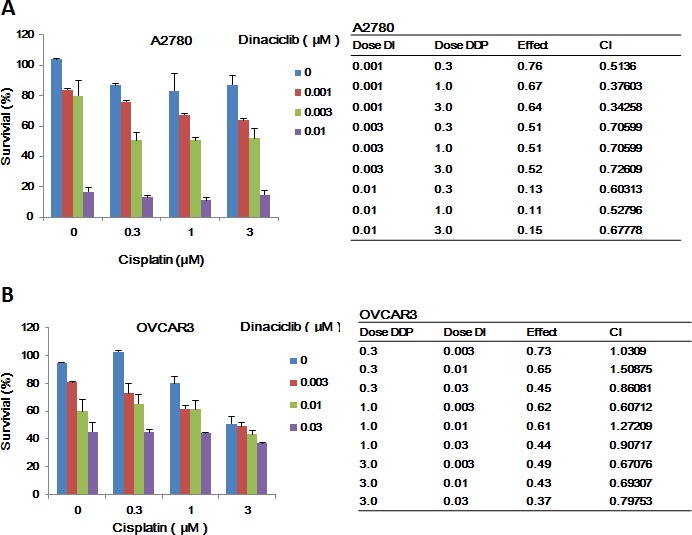
Dinaciclib synergized with cisplatin to inhibit the growth of ovarian cancer cells *in vitro* A2780 (**A**) and OVCAR3 (**B**) cells were treated with the indicated concentrations of dinaciclib and cisplatin.for 72 h, and cell survival was detected by MTT assay. The data were analyzed by CompuSyn software with the results shown as growth histogram, dose-effect curve, CI values and normalized isobologram.

Combination therapy is the main mode of cancer chemotherapy because of its significant advantages such as slower development of drug resistance and lower treatment failure rate [22]. Cisplatin currently is the first-line chemotherapeutic drug for ovarian cancer in clinic. To check the combined effects of dinaciclib and cisplatin on ovarian cancer cells, cell survival was detected by MTT assay. After combined treatment with dinaciclib (0.001, 0.003, 0.01 and 0.03 μM) and cisplatin (0.3, 1, 3, 10 and 30 μM), the survival of cells was significantly reduced in compared with dinaciclib or cisplatin alone treatment in both A2780 and OVCAR3 cells. Almost all CI values of combination were <1 (Figure 5A and 5B), suggesting that the antigrowth effects of dinaciclib combined with cispaltin in the indicated ovarian cancer cells was synergistic rather than additive.

### Dinaciclib synergized with cisplatin to induce cell cycle arrest and apoptosis in ovarian cancer cells

**Figure 6:**
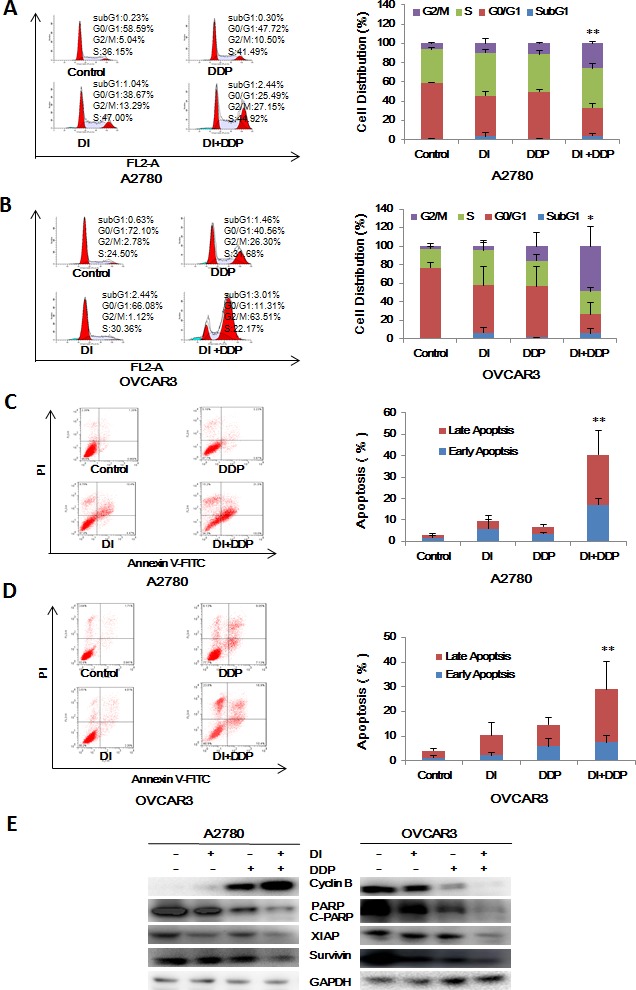
Dinaciclib synergized with cisplatin to induce cell cycle arrest and apoptosis in ovarian cancer cells A2780 (**A**, **C**) and OVCAR3 (**B**, **D**) cells were treated with 0.01 μM dinaciclib, 3 μM cisplatin alone or combination for 48 h. The distribution of cell cycle was detected by FCM with PI staining. The percentages of subG1, G1/G0, S, G2/M phase were calculated using ModFit LT 3.0 software. The apoptosis was detected by FCM Annexin V/PI staining. The proportions of Annexin V+/PI− and Annexin V+/PI+ cells indicated the early and late stage of apoptosis. The protein expression was examined by Western blot after lysing cells, and GAPDH was used as loading control. The representative charts, quantified results and Western blot results (**E**) of three independent experiments were shown. DI: Dinaciclib; DDP: Cisplatin. *P < 0.05 *vs.* corresponding control.

To further estimate the synergistic effects of dinaciclib and cisplatin on ovarian cancer cells, cell cycle distribution and apoptosis were examined by FCM with PI and Annexin V/PI staining. In A2780 cells, either dinaciclib or cisplatin alone treatment induced the accumulation in G2/M phase and reduction in G0/G1 phase of cell population, and co-treatment with dinaciclib and cisplatin induced the more significant accumulation in G2/M phase and reduction in G0/G1 phase of cell population (Figure 6A). While in OVCAR3 cells, dinaciclib alone treatment induced the accumulation in S phase and reduction in G0/G1 phase of cell population, and cisplatin alone treatment induced the accumulation in G2/M phase and reduction in G0/G1 phase of cell population, and co-treatment with dinaciclib and cisplatin induced the dramatical accumulation in G2/M phase and reduction in G0/G1 phase of cell population (Figure 6B). Additionally, either dinaciclib or cisplatin alone treatment caused mild apoptosis, and co-treatment with dinaciclib and cisplatin caused significant apoptosis in both cells (Figure 6C and 6D). Moreover, the related proteins of cell cycle and apoptosis were detected by Western blot to investigate the molecular mechanism of the synergistic effects of dinaciclib and cisplatin on cell cycle and apoptosis in ovarian cancer cells. As shown in Figure 6E, either dinaciclib or cisplatin alone treatment moderately increased the protein levels of C-PARP and decreased the protein levels of XIAP and survivin, and co-treatment with dinaciclib and cisplatin significantly increased the protein levels of C-PARP and decreased the protein levels of XIAP and survivin. Interestingly, the protein levels of Cyclin B were increased moderately in either dinaciclib or cisplatin alone group and significantly in the combination group of dinaciclib with cisplatin in A2780 cells, but deceased in OVCAR3 cells.

### Dinaciclib synergized with cisplatin to inhibit the subcutaneous xenograft growth of ovarian cancer in nude mice

**Figure 7:**
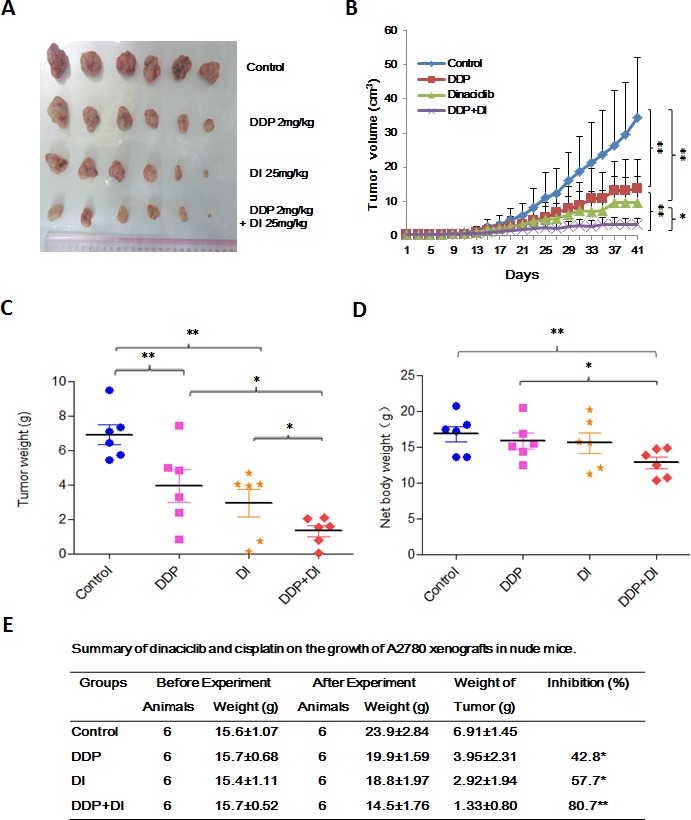
Dinaciclib synergized with cisplatin to inhibit the subcutaneous xenograft growth of ovarian cancer in nude mice Each mouse was injected subcutaneously with A2780 cells (2 × 10^6^ in 100 μl of medium) under the shoulder. When the subcutaneous tumors were approximately 0.3 × 0.3 cm^2^ (two perpendicular diameters) in size, mice were randomized into four groups, and were injected intraperitoneally with vehicle alone (20% hydroxypropyl-β-cyclodextrin), dinaciclib alone (25 mg/kg), cisplatin alone (2 mg/kg), or a combination of dinaciclib and cisplatin every four days. The body weights of mice and tumor volume were recorded. The mice were anaesthetized after experiment, and tumor tissue was excised from the mice and weighted. The original tumors (**A**), tumor volume (**B**), tumor weight (**C**), net body weight (without tumor) (**D**) and summary data (**E**) were shown. The values presented are the means ± SD for each group. DI: Dinaciclib; DDP: Cisplatin. *P < 0.05 and **P < 0.01 *vs.* corresponding control.

To test the synergistic antitumor effects of dinaciclib and cisplatin *in vivo*, we generated the subcutaneous xenograft tumor models by transplanting A2780 cells into nude mice. As shown in Figure 7A and 7B, compared with dinaciclib or cisplatin alone treatment, co-treatment with dinaciclib and cisplatin clearly inhibited the tumors growth by reducing the volume and weight of A2780 tumors. The inhibition rates of tumor growth in the combined group were 80.7%, which were significantly higher than those in cisplatin (42.8%) or dinaciclib (57.7%) alone group (Figure 7C). Nevertheless, the net body weights (without tumor) of mice in the combined group were lower than those in control group, suggesting co-treatment of dinaciclib and cisplatin at the indicated dose caused certain side effects in mice (Figure 7B).

## DISCUSSION

In this study, we show that dinaciclib has the potent ability to induce cell growth inhibition, cell cycle arrest and apoptosis with the increased intracellular ROS levels in human ovarian cancer cells. Interestingly, treatment with dinaciclib leads to the different distribution of cell cycle in our tested two ovarian cancer cells, which accompanies the distinct protein expressions of some important cell cycle related protein such as Cdk2 and cyclin E. Thus, the cell cycle responses of ovarian cancer cells to dinaciclib are variable and may be due to cellular genotype. Previous publications demonstrate that dinaciclib is able to promote apoptosis in multiple types of cancers [15, 23-28]. In our case, dinaciclib also triggers apoptosis in ovarian cancer cells with the reduced protein expressions of anti-apoptotic proteins including Mcl-1, XIAP, survivin, etc. Notably, Mcl-1 and Bcl-xL levels in solid tumors are the predictive biomarkers for dinaciclib induced apoptosis and antiumor response [29], and dinaciclib potently inhibits Mcl-1 to induce durable apoptosis in aggressive MYC-driven B-cell lymphoma [30]. Therefore, detection of Mcl-1 levels in tumor tissues may be a valuable strategy to identify cancer patients most probably respond to dinaciclib.

The intracellular ROS plays an important role in regulating various cell physiological process such as growth, differentiation, death, and so on [31]. ROS can change the cellular redox condition, induce DNA damage, and influence the activities of oncogene or tumor suppressor, thereby involving in the initiation and progression of cancer [32]. Cancer cells usually exhibit increased basal levels of intrinsic oxidative stress, and a further elevation of intracellular ROS above the toxic threshold level may result in cell death [33]. Accordingly, this oxidative shift may cause cancer cells susceptible to chemotherapeutic agents that work by amplifying ROS generation [34]. However, apoptosis induced by some stimuli is independent of ROS generation such as etoposide-induced apoptosis in B lymphoma cells [35], emodin-induced apoptosis in promyeloleukemic cells [36]. In addition, several anti-oxidant agents including vitamin E are able to induce apoptosis in cancer cells [37]. Similar to other CDK inhibitors flavopiridol and roscovitine [38, 39], dinaciclib also can enhance the intracellular ROS levels, and NAC pretreatment dramatically blocks ROS generation but partially rescues dinaciclib-induced apoptosis in the current study. Our results suggest that dinaciclib is able to prompt both ROS dependent and independent apoptosis in ovarian cancer cells. Cisplatin is the first member of platinum-containing family anticancer drugs, which also includes carboplatin and oxaliplatin, to trigger cancer cells death by binding to and causing crosslinking of DNA, and now is used to treat various types of cancers including ovarian cancer, lung cancer, sarcomas, germ cell tumors, etc [40]. It has been reported that cisplatin combined with flavopiridol is highly synergistic to kill cancer cells in the preclinical settings [41], and this combination is active for cancer patients based on the encouraging results of two clinical trial [42, 43]. Interestingly, compared with parental cells, ovarian cancer cells resistant to flavopiridol and cisplatin showed the increased expressions of CDK1, cyclin D3 and Rb, decreased expressions of cyclin B, and equal expressions of cyclin A, cyclin E, CDK2, CDK4 [44]. Recently, it has demonstrated that increased DNA ploidy is associated with resistance to dinaciclib in ovarian cancer cells [45]. Dinaciclib has shown a synergistic anticancer effect when combined with other chemotherapeutic agents including gemcitabine, vinblastin and NVP-AUY922 in pancreatic cancer, leukemia and osteosarcoma, respectively [46-48]. Additionally, dinaciclib in combination with rituximab is well tolerated and has favorable clinical activity in relapsed/refractory chronic lymphocytic leukemia patients [49]. In our study, co-treatment with dinaciclib and cisplatin synergistically induces cell growth inhibition, cell cycle arrest and apoptosis in ovarian cancer cells, and further suppresses the subcutaneous xenograft growth of ovarian cancer in nude mice. It has reported that dinaciclib is metabolized by CYP3A4 [50], and cisplatin is the substrate of SLC22A1, SLC22A2, SLC31A1, ABCC2, ABCC4, ATP7B and SLC47A1 transporters [51]. Whether dinaciclib could affect the pharmacokinetic, pharmacodynamic and clinical outcome of cisplatin in ovarian cancer patients remains to be determined.

In conclusion, our study not only demonstrates that monotherapy with dinaciclib is highly active in inducing ovarian cancer cell cycle arrest and apoptosis *in vitro* and *in vivo*, but also shows that combinational treatment with dinaciclib and cisplatin synergistically inhibit cells growth in preclinical models of ovarian cancer. Dinaciclib in combination with cisplatin shows potently synergistic anticancer effect, indicating this beneficial combinational therapy may be a promising strategy for treatment of ovarian cancer.

## MATERIALS AND METHODS

### Cell culture and reagents

Human ovarian cancer cell lines A2780, OVCAR3, SKOV3, HO8910, HO8910PM, ES-2 and normal embryonic kidney cell line HEK293T were cultured in Dulbecco's modified Eagle's medium (DMEM) containing 10% fetal calf serum (FBS), penicillin (100 U/ml) and streptomycin (100 ng/ml) in a humidified incubator at 37°C with 5% CO_2_. Dinaciclib and cisplatin were ordered from ApeBio and Qilu Pharmaceutical, respectively. N-acetly-L-cysteine (NAC) and dihydroethidium (DHE) were purchased from Sigma-Aldrich. Anti-pRb pS807/S811 (9308), Anti-PARP (9542), Anti-Mcl-1 (4572), Anti-Survivin (2808), Anti-XIAP (2045) and Anti-β-Catenin (9582) antibodies were from Cell Signaling Technologies. Anti-CDK2 (SC-163), Anti-CDK5 (SC-173), Anti-Cyclin A (SC-596), Anti-Cyclin E (SC-481), Anti-HSP90 (SC-13119), Anti-MDM2 (SC-965), and Anti-Raf-1 (SC-133) antibodies were from Santa Cruz Biotechnology. Anti-p27 (610241), Anti-Cyclin B (610219) and Anti-CDK1 (610037) antibodies were from BD Bioscences. Anti-CDK9 (2496-1) was from Epitomics. Anti-β-Actin (LK9001T) and Anti-GAPDH (LK9002T) antibodies were from Tianjin Sungene Biotech.

### Cell viability assay

Cells were firstly seeded into a 96-well plate at a density of 5000 cells per well, and incubated with drugs in three parallel wells for 72 h. Then 3-(4, 5-dimethylthiazolyl-2)-2, 5-diphenyltetrazolium bromide (MTT) was added to each well at a final concentration of 0.5 mg/ml. After incubation for 4 h, formazan crystals were dissolved in 100 ml of DMSO, and absorbance at 570 nm was measured by plate reader. The concentrations required to inhibit growth by 50% (IC_50_) were calculated from survival curves using the Bliss method [52, 53]. For drug combination experiments, cells were co-treated with different concentrations of dinaciclib and cisplatin for 72 h. The data were analyzed by CompuSyn software with the results showed as combination index (CI) values according to the median-effect principle, where CI <1, =1, and >1 indicate synergism, additive effect, and antagonism, respectively [21, 54].

### Cell cycle assay

Cells were harvested and washed twice with cold phosphate-buffered saline (PBS), then fixed with ice-cold 70% ethanol for 30 min at 4°C. After centrifugation at 200 × g for 10 min, cells were washed twice with PBS and resuspended with 0.5 ml PBS containing PI (50 μg/ml), 0.1% Triton X-100, 0.1% sodium citrate, and DNase-free RNase (100 μg/ml), and detected by FCM after 15 min incubation at room temperature in the dark. Fluorescence was measured at an excitation wavelength of 480 nm through a FL-2filter (585 nm). Data were analyzed using ModFit LT 3.0 software (Becton Dickinson).

### Apoptosis assay

Cell apoptosis was evaluated with flow cytometry (FCM) assay. Briefly, cells were harvested and washed twice with PBS, stained with Annexin V-FITC and propidium iodide (PI) in the binding buffer, and detected by FACSCalibur FCM (BD, CA, USA) after 15 min incubation at room temperature in the dark. Fluorescence was measured at an excitation wave length of 480 nm through FL-1 (530 nm) and FL-2 filters (585 nm). The early apoptotic cells (Annexin V positive only) and late apoptotic cells (Annexin V and PI positive) were quantified.

### Reactive oxygen species (ROS) assay

Cells were incubated with 10 μM of DHE for 30 min at 37°C, and observed under fluorescence microscope (Olympus, Japan) immediately after washing twice with PBS. Five fields were taken randomly for each well.

### Western blot analysis

Cells were harvested and washed twice with cold PBS, then resuspended and lysed in RIPA buffer (1% NP-40, 0.5% sodium deoxycholate, 0.1% SDS, 10 ng/ml PMSF, 0.03% aprotinin, 1μM sodium orthovanadate) at 4°C for 30 min. Lysates were centrifuged for 10 min at 14,000 × g and supernatants were stored at −80 °C as whole cell extracts. Total protein concentrations were determined with Bradford assay. Proteins were separated on 12% SDS-PAGE gels and transferred to polyvinylidene difluoride membranes. Membranes were blocked with 5% BSA and incubated with the indicated primary antibodies. Corresponding horseradish peroxidase-conjugated secondary antibodies were used against each primary antibody. Proteins were detected using the chemiluminescent detection reagents and films.

### *In vivo* tumor xenograft assay

Balb/c nude mice were obtained from the Guangdong Medical Laboratory Animal Center and maintained with sterilized food and water. Six female nude mice with 5 weeks old and around 15 g weight were used for each group. Each mouse was injected subcutaneously with A2780 cells (2 × 10^6^ in 100 μl of medium) under the shoulder. When the subcutaneous tumors were approximately 0.3 × 0.3 cm^2^ (two perpendicular diameters) in size, mice were randomized into four groups, and were injected intraperitoneally with vehicle alone (20% hydroxypropyl-β-cyclodextrin), dinaciclib alone (25 mg/kg), cisplatin alone (2 mg/kg), or a combination of dinaciclib and cisplatin every four days. The body weights of mice and the two perpendicular diameters (A and B) of tumors were recorded. The tumor volume (V) was calculated according to the formula:

$$\text{v}=\frac{\text{π}}{6}{\left(\frac{\text{A+B}}{2}\right)}^{3}$$

The mice were anaesthetized after experiment, and tumor tissue was excised from the mice and weighted. The rate of inhibition (IR) was calculated according to the formula:

$$\text{IR}=1-\frac{\text{Mean tumor weight of experimental group }}{\text{Mean tumor weight of control group}}\times 100\%$$

### Statistical analysis

All results are expressed as mean ± standard deviation (SD). Statistical analysis of the difference between treated and untreated groups was performed with Student's t-test. Values of *P* < 0.05 were considered as significant differences.
